# A quantitative analysis of microbial community structure-function relationships in plant litter decay

**DOI:** 10.1016/j.isci.2022.104523

**Published:** 2022-06-03

**Authors:** Bonnie Waring, Anna Gee, Guopeng Liang, Savannah Adkins

**Affiliations:** 1 Grantham Institute on Climate Change and the Environment and the Georgina Mace Centre for the Living Planet, Imperial College London, London SW7 2BU, UK; 2 Department of Forest Resources, University of Minnesota, St. Paul, 55108 MN, USA; 3 Department of Biology, Utah State University, Logan, 84321 UT, USA

**Keywords:** Microbiology, Plant biology, Soil ecology

## Abstract

Soil microbes play a central role in ecosystem element cycling. Yet a central question in microbial ecology remains unanswered: to what extent does the taxonomic composition of soil microbial communities mediate biogeochemical process rates? In this quantitative review, we explore the mechanisms that lead to variation in the strength of microbial community structure-function relationships over space and time. To evaluate these mechanisms, we conduct a meta-analysis of studies that have monitored the decomposition of sterilized plant litter inoculated with different microbial assemblages. We find that the influence of microbial community composition on litter decay is pervasive and strong, rivalling in magnitude the influence of litter chemistry on decomposition. However, no single environmental or experimental attribute was correlated with variation in the inoculum effect. These results emphasize the need to better understand ecological dynamics within microbial communities, particularly emergent features such as cross-feeding networks, to improve predictions of soil biogeochemical function.

## Graphical abstract

**Figure:**
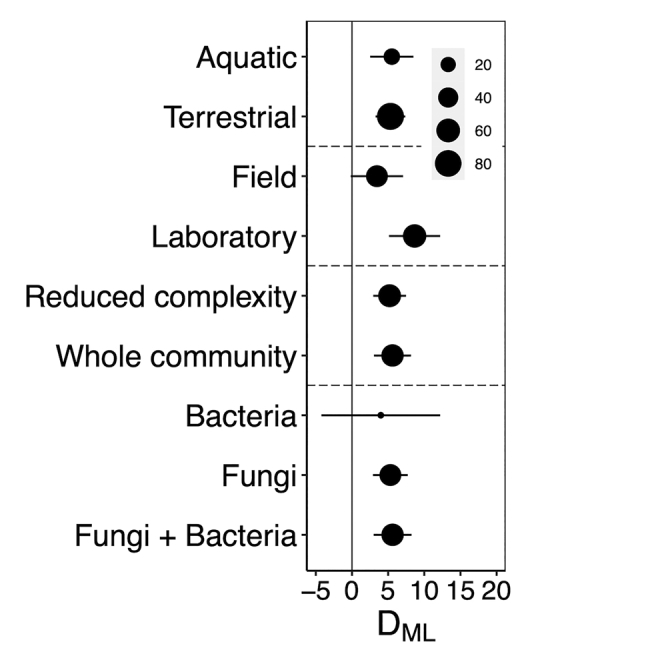

## Introduction

Microbes in soils and sediments govern the fluxes of carbon, nitrogen, and other elements through ecosystems and thereby regulate global biogeochemical cycles. Although decomposer microbes are biodiverse and ubiquitous, we struggle to answer a basic question about their ecology: does the identity and abundance of different microbial species (i.e., the structure of microbial communities) control soil process rates (i.e. function)? Hundreds of researchers have assessed microbial structure-function relationships in soils and sediments, either directly through manipulative experiments or indirectly by tracking shifts in microbial communities alongside corresponding changes in biogeochemistry. These studies have significantly advanced our understanding of soil microbial biogeography (Crowther et al., 2019), metabolism (Yao et al., 2018), and influence over biogeochemical cycles (Schimel and Schaeffer, 2012; Hartman et al., 2017). Some experiments demonstrate conclusively that variation in microbial community structure drives changes in ecosystem properties (e.g., Averill et al., 2016; Strickland et al., 2009). Others show limited correspondence between the composition of microbial communities and their functions (e.g., Kemmitt et al., 2008; Wertz et al., 2007; Wertz et al., 2006). To guide research efforts, improve predictive biogeochemical models, and advance our fundamental understanding of microbial ecology, we must identify *why* different studies show variation in the strength of the microbial community structure-function relationship.

Here, we review the factors, both methodological and ecological, that have complicated efforts to identify directional relationships between the composition of microbial communities and their functions. We discuss how soil microbial community structure-function relationships change as a function of the ecological, temporal, and environmental distances over which they are examined. To illustrate these context dependencies and assess the strength of microbial community influences on a key ecosystem process (decomposition), we present a meta-analysis of studies that have assessed community structure-function relationships by inoculating sterilized organic matter with specific microbial assemblages. As described in more detail in the following sections, such studies were designed to isolate microbial effects on the key ecosystem process of decomposition and therefore are ideally suited to address community structure-function relationships. Finally, we consider the implications of our findings for our ability to predict biogeochemical patterns and processes.

### Challenges in the quantification of soil microbial community structure-function relationships

The importance of soil microbial community composition for ecosystem function is a central research theme in microbial ecology. Many excellent literature reviews have synthesized the enormous body of literature relevant to this topic (e.g. Schimel and Schaeffer, 2012; Crowther et al., 2019; Graham et al., 2016; Morris et al., 2020; Rocca et al., 2015). Each of them elaborates some of the major challenges that impede our understanding of soil microbial communities—the magnitude of microbial diversity, a high yet unpredictable degree of functional redundancy, and complex spatial dynamics in soils—discussed in detail below.

#### Experimental approaches to examine links between microbial diversity and ecosystem processes

A gram of soil can contain hundreds or thousands of unique bacterial and fungal taxa that can rapidly respond to—and alter—conditions in the abiotic environment. This diversity presents a formidable challenge in isolating the direction of causality in microbial structure-function relationships. Studies seeking to isolate microbial influence over ecosystem processes generally employ one of a few standard approaches: biodiversity-ecosystem function experiments, common garden experiments, or reciprocal transplants (Reed and Martiny, 2007). Classic biodiversity-function experiments, in which microbial communities are assembled at various levels of species richness, can isolate the effects of taxonomic or functional diversity on ecosystem processes. Results of these studies have been decidedly mixed. For example, one set of experiments used serial dilution of whole-soil microbial inocula to establish a steep gradient of taxonomic diversity; carbon mineralization and nitrogen cycling were unaffected by the loss of 99.9% of bacterial species (Wertz et al., 2006, 2007). Yet a separate experiment with a very similar serial dilution design found that rates of potential denitrification dropped sharply with a decrease in denitrifier diversity (Philippot et al., 2013). Similarly, a diversity manipulation with 72 culturable bacterial species found that carbon mineralization rates decreased with declining taxonomic diversity of artificial assemblages (Bell et al., 2005). What might account for this cross-study variation? Biodiversity and ecosystem process rates often exhibit positive but saturating relationships, especially when examined over small spatial and temporal scales (Qiu and Cardinale, 2020). Different experiments may have variable success in reducing diversity below the critical threshold where diversity and function are strongly positively correlated. To our knowledge, however, no study has explicitly assessed whether the strength of the microbial diversity-function relationship hinges on the length or steepness of the diversity gradient.

Common garden experiments (in which different microbial communities are incubated in the same environment) or reciprocal transplant experiments (in which different microbial assemblages are incubated across multiple environments) can illustrate interactions among community composition, characteristics of the environment, and function. As is true for biodiversity-function experiments, the results of such studies provide conflicting insights on the role of microbial community composition in mediating soil functions. Multiple experiments have inoculated litter or soil with whole-soil microbial inocula isolated from different points along steep climate or resource gradients, then monitored carbon mineralization under controlled environmental conditions. In some cases, the composition of the microbial inoculum has strong impacts on carbon cycling rates (Glassman et al., 2018; Hawkes et al., 2017; Keiser et al., 2011). In other such experiments, however, the effects of microbial community composition on functioning are absent or dissipate over time (Martiny et al., 2017; Rousk et al., 2013). These declines in effect size might be explained by immigration of taxa from the surrounding environment, which would diminish both compositional and functional dissimilarity among microbial communities in common garden experiments. Some microbial communities show high resistance to invasion by new taxa (Waring and Hawkes, 2018), but it is unclear what ecological or environmental factors govern this resistance. We also lack a rigorous evaluation of how microbial inoculum effects on ecosystem processes change through time and the factors that might mediate these temporal dynamics.

It is even more challenging to unravel community structure-function relationships in experimental contexts that do not directly manipulate soil microbial communities. For example, many studies have shown that soil microbial community composition responds to elevated CO_2_ (Hu et al., 1999), soil warming (Melillo et al., 2017), changes in rainfall (Hawkes et al., 2017), and nutrient deposition (Ramirez et al., 2010). However, it is much more difficult to assess the extent to which community shifts subsequently drive changes in soil biogeochemistry. A recent meta-analysis found that microbial community characteristics usually did not enhance the ability to predict patterns in carbon and nitrogen cycling, although there were some exceptions to this rule (Graham et al., 2016). Similarly, in real-world environments, the abundances of microbial functional genes are not strongly correlated with the biogeochemical processes those genes mediate (Rocca et al., 2015). These tenuous structure-function linkages may result from high functional redundancy, complex emergent properties in community metabolic networks, or spatial and temporal separation between microbes and their organic matter substrate, as discussed later.

#### Functional redundancy and context dependency in microbial community structure-function relationships

Linkages between microbial community structure and function may appear inconsistent because these relationships are highly sensitive to the function that is being quantified. There is likely to be a large overlap in the fundamental niches of many microbial taxa, generating a high degree of functional redundancy. Schimel et al. (1995) confronted this issue by distinguishing between “narrow” and “broad” biogeochemical processes. Narrow functions, such as denitrification, are carried out by a small subset of taxa, within which community structure-function relationships may be easier to discern. However, some of the most critical biogeochemical functions (e.g. CO_2_ production) are carried out by the vast majority of the microbial community. The relative abundance of different microbial taxa may still influence broad functions, but these relationships are likely to be weaker and dependent upon environmental conditions. For example, the identity of the microbial taxa that actively respire CO_2_ may vary with fluctuations in soil moisture (Placella et al., 2012) or temperature (Oliverio et al., 2017). Despite these context dependencies, even within a fairly specific range of environmental conditions, there may be dozens or hundreds of taxa that are capable of carrying out broad functions such as nutrient mineralization or CO_2_ respiration. Theoretically, this should buffer process rates against shifts in microbial community structure (Louca et al., 2018). Surprisingly, however, there does not appear to be a straightforward relationship. For example, in one of the bacterial diversity manipulations described earlier, broad and narrow functions were equally insensitive to the loss of microbial taxa (Wertz et al., 2006). Conversely, some of the strongest relationships between ecosystem process rates and microbial community structure are observed for soil respiration (e.g. (Hawkes et al., 2017)), the most general of soil microbial functions.

Although processes such as mineralization of carbon, nitrogen, and phosphorus are carried out by most soil decomposers, these fluxes emerge from thousands of biochemical reactions that break down complex plant biomolecules. Not all members of the microbial community can carry out the full suite of extracellular and intracellular reactions that ultimately convert plant biomass into CO_2_ or inorganic nutrients; in other words, even “broad” processes such as respiration reflect a multitude of underlying “narrow” processes. For this reason, the chemical composition of organic matter influences its mineralization to CO_2_ by the soil microbial community, as most microbial taxa appear to specialize in either recalcitrant or labile substrates (Goldfarb et al., 2011; Fierer et al., 2007; Mcguire et al., 2010). The decay of some biomolecules, such as lignin, is taxonomically restricted to the fungi and bacteria that can produce the necessary decompositional enzymes (Brown and Chang, 2014; De Boer et al., 2005). Thus, CO_2_ mineralization from decomposing litter may be more rapid when microbial communities are characterized by a high “functional breadth,” with diverse taxa that can participate in the depolymerization of a large suite of macromolecules (Osburn et al., 2022).

Decay rates may also be influenced by the interaction between microbial community composition and plant tissue chemistry, a phenomenon best studied in the context of “home-field advantage”; this refers to scenarios where decay rates are faster when microbial communities are paired with litter from their “home” ecosystem or habitat and slower when decomposers encounter chemically novel substrates (Ayres et al., 2009; Strickland et al., 2009). Home-field advantage effects tend to be stronger when “home” versus “away” substrates are more dissimilar (Veen et al., 2015), which implies that they emerge from local adaptation of microbes to litter type.

The effects of microbial communities on belowground processes may also be context dependent because of interactions within microbial communities. Qiu and Cardinale (2020) describe several mechanisms by which increasing diversity within a guild enhances function: through niche complementarity, leading to more complete exploitation of resources; through facilitation, which enhances community-level resource use and growth; or through selection effects, whereby the probability of identifying a functionally unique decomposer increases with the number of species included in an experiment. Within decomposer communities specifically, it is well known that competition between microbial taxa can strongly influence the rate at which organic matter is decomposed (Buchkowski et al., 2017). However, facilitative interactions may lead to the emergence of cross-feeding networks (Pascual-García et al., 2020), whereby the byproducts of one taxon’s metabolism are further metabolized by another taxon (Smith et al., 2019). Ultimately, such interactions can enlarge the realized niche of species present in a consortium. The self-organization of such cross-feeding networks appears to be ubiquitous across microbial communities and is a major factor in maintaining their high biodiversity (Goldford et al., 2018). However, this also means that ecosystem process rates are an emergent property of interactions within the microbial community; thus, the presence or absence of an individual taxon may not carry much information. Moreover, context dependencies in community assembly could lead to positive correlations between microbial diversity and the *variability* of process rates, reflecting the multiple alternative trajectories by which functionally distinct cross-feeding networks can develop. However, there have been few, if any, systematic attempts to assess whether the ecological distances across different microbial communities correspond to their functional dissimilarity.

#### Spatial and temporal heterogeneity in soils and their influence on microbial structure-function relationships

Even if a particular taxon could potentially exert a large influence on some ecosystem process, its presence in the soil environment may or may not influence actual biogeochemical reactions. Soil is probably the most heterogeneous environment on earth: although a gram of soil may contain billions of microbial cells, they occupy less than 0.001% of the soil surface area (Young and Crawford, 2004). Microbes require access to the organic matter they transform; if a microbial cell and its substrate are separated in space or time, then a given reaction will not occur. Moreover, once a microbe has acted upon its substrate, the constituent elements may be lost from the soil system through the production of trace gases, or incorporated into the biomass, where they can be subject to further processing by the microbial community. In recognition of these points, Schimel and Schaeffer (2012) provide a framework to distinguish between the *rate* of a given process (i.e., the amount of time needed for complete decomposition of litter) versus the *fate* of the organic matter involved in that process (i.e., immobilization in microbial biomass and residues versus mineralization to CO_2_). If the composition of microbial communities affects process rates but not organic matter fates, then the influence of microbial community processes is negligible when viewed in the long term. By contrast, if microbial anabolism is an important control over the dynamics of long-term organic matter stabilization, then the metabolic capacities of a given microbial assemblage may have long-lasting impacts.

#### To what extent does the structure of microbial communities influence their functions? A meta-analysis of decomposition experiments as a case study

Given the magnitude of microbial diversity and the heterogeneity of the soil environment, it is unsurprising that we still struggle to make specific predictions about the role of soil microbial community composition in driving biogeochemical processes. In some cases, microbes have a strong influence on diverse processes such as litter decay (Strickland et al., 2009), soil respiration (Hawkes et al., 2017), or nitrogen transformations (Philippot et al., 2013); in other instances, these linkages are tenuous or absent. How can we predict when and where information about microbial community structure is important for understanding ecosystem responses to environmental change?

To explore the issues raised in this review, we present a meta-analysis of litter decomposition experiments wherein sterilized litter was inoculated with at least two distinct microbial assemblages. We specifically focus on litter decomposition experiments because they avoid the complex issue of soil spatial heterogeneity, as microbes have unrestricted access to organic matter in the litterbag environment. We quantified the strength of microbial community inoculum effects on ecosystem process rates (decomposition) by examining variability in the decay rates of standard substrates across treatment with different inocula. This approach permits us to address many of the unresolved questions described earlier in a quantitative way. Specifically, to assess whether the steepness of taxonomic diversity gradients influences structure-function relationships, we compared effect sizes between experiments with whole-soil versus reduced-complexity inoculum and across studies that generated different taxonomic diversity gradients. To assess how microbial community effects on process rates change over time, we assessed effect sizes in relation to the duration of decomposition. We also examined the relationship between litter quality and microbial influence over decay. Finally, where information about microbial community structure was available, we probed the relationships between (dis)similarity of microbial communities and their functional rates. Our dataset enabled us to test four key hypotheses: (H1) experiments with shorter diversity gradients (i.e., a smaller number of taxa in the most diverse treatment) should demonstrate larger inoculum effects on decomposition, because diversity-function relationships saturate at higher levels of diversity. (H2) Inoculum effects should change with experimental duration, reflecting temporal divergence of microbial communities via formation of cross-feeding networks, species loss, or immigration. (H3) Inoculum effects should be more pronounced on recalcitrant litter types, as decomposition of such nutrient-poor, lignin-rich litters is carried out by a more taxonomically restricted group of organisms. (H4) Dissimilarity in decay rates across inoculum treatments can be explained by variation in microbial community composition, because communities that exhibit a higher overlap in species composition are also more functionally alike.

### Quantifying microbial community structure-function relationships through a meta-analysis

#### Experimental design and methods

On 2 September 2021, we searched Web of Science using the terms “decomp∗ AND litter AND inocul∗”; we did not search for “SOM” or “mineralization,” as we wished to avoid studies that quantified the decomposition of soil organic matter (introducing spatial constraints on microbial access to substrate). This search returned 241 papers; after eliminating those that did not use at least two different microbial “community” treatments, or that did not report mass loss data, we retained 36 publications for meta-analysis (Table S1). We included studies conducted in the laboratory or in the field, so long as the material decomposed was initially sterilized and re-inoculated with soil bacteria and/or fungi before deployment. Our search terms captured studies in two broad groups: first, those which inoculated litter with specific bacterial or fungal strains, either alone or in defined combinations (so-called “reduced complexity” inoculum experiments); second, studies where sterile litter was inoculated with whole-soil inocula containing the full complement of bacterial and fungal species that would be found in the soil/sediment environment (“whole community” inoculum experiments). We recorded the location, ecosystem type, and climatic conditions where each field experiment was conducted; whether the inoculum used consisted of bacteria, fungi, or both; the source of the inoculum used; the type of litter being decomposed and its chemical attributes (C:N ratio and lignin content); the timing of each mass loss measurement (i.e., the number of days since decomposition began); the number of replicates (*N*) in each treatment group; the response variable (decay rate) and the SE of decay rate in each treatment. Finally, as all publications using a “reduced-complexity” inoculum approach controlled the number of taxa in each inoculum treatment level, we recorded the length of the diversity gradient as the maximum bacterial or fungal species richness for each of these studies (studies used monocultures as the minimum diversity treatment). In other words, each study was associated with the number of taxa combined in the highest-diversity inoculum treatment.

For each study, we calculated an inoculum effect size metric: the coefficient of variation (CV or SD divided by the mean) of mass loss across inoculum treatments in each study. Because CV is a unitless quantity, analyzing this response metric allowed us to compare patterns across all studies, regardless of the method with which decomposition was quantified. When an individual study quantified mass loss at different time points, or applied inoculum treatments to different types of litter substrate, *CV* was calculated independently for each unique combination of litter type and time point. For example, if an experiment added four different microbial inocula to two different types of litter and monitored decomposition at 6, 12, and 24 months, we would calculate six estimates of CV, corresponding to variability in mass loss across inoculum treatments within each litter type at each of the three time points. Therefore, our dataset yielded 141 unique observations of effect size across the 36 studies included in the meta-analysis. For the six studies that incorporated different litter treatments, we also calculated CV among these; i.e., we examined the pairwise distance in mass loss rates among different types of leaf or root litter, averaging across microbial inoculum treatments.

To translate our CV into an estimate of the biological effect size of the inoculum treatments, we also calculated *D,* or the mean pairwise Euclidean distance among decay rates in different inoculum treatments. Twenty-five studies reported litter mass loss as a percentage of initial mass and 11 reported evolution of CO_2_ from the litter (mg CO_2_-C g^−1^ litter). As these units are not directly comparable, we calculated and analyzed *D* for the two types of response variable (henceforth, *D*_*M*L_ and *D*_*CO2*_) in separate models. The results of these models provide a direct estimate of the biogeochemical effect size of the inoculum treatments. For example, a *D*_*ML*_ of 2.0 would indicate that the difference in mass loss among different inoculum treatments corresponds to 2% of initial litter mass.

We conducted multilevel mixed-effects meta-analyses to examine the effects of various categorical predictors on our effect size metrics. For each of these models, study identity was used as a grouping factor to control for nonindependence of observations from the same experiment (i.e., experiments that monitored inoculum treatments at multiple time points). We calculated the within-study variance associated with each effect size (*CV, D*_*ML*_ and *D*_*CO2*_) using SD of mass loss within each inoculum treatment following (Borenstein et al., 2009). Significant effect size outliers were excluded before analyses. All analyses were conducted in the R package *metafor* (Viechtbauer, 2010).

Finally, we quantified the relationship between microbial community dissimilarity and functional dissimilarity in mass loss across inoculum treatments. Bacterial and fungal community composition data were recorded as scores on the first and second axes of an NMDS or PCA plot. Because numeric axes values in these plots are essentially meaningless (only the relative distance among data points is important), we did not attempt to compare patterns of community dissimilarity *across* studies. Rather, we evaluated the correspondence between ordination scores and mass loss values *within* a study. Essentially, for each experiment, we were testing the hypotheses that more compositionally distinct inoculum treatments would tend to have more dissimilar rates of decomposition than inoculum treatments that shared more microbial taxa in common. To evaluate this hypothesis, for each study, we generated a distance matrix for ordination scores and one for mass loss values, then used a Mantel test to evaluate the correspondence between these matrices. Only six of the publications included in this meta-analysis provided microbial community data; of these, four provided information for both fungal and bacterial communities (analyzed with separate Mantel tests) and two reported bacterial community data only.

## Results

**Figure 1:**
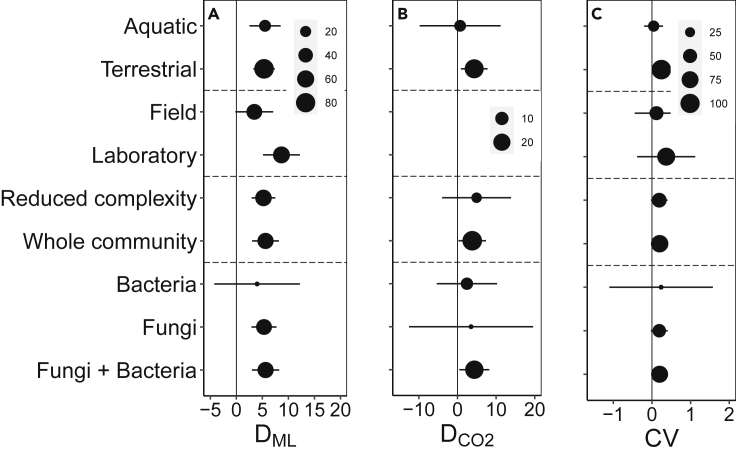
Effect sizes and 95% confidence intervals for subgroups within the meta-analysis The metrics of effect size are (A) *D*_*ML*_ (mean pairwise distance in mass loss, % of initial litter mass), (B) *D*_*CO2*_ (mean pairwise distance in CO2 mineralization, mg CO_2_-C g^−1^ litter), and (C) *CV* (coefficient of variation in mass loss, unitless). Symbol sizes indicate the number of observations within each subgroup.

Across all observations included in the dataset, litter mass loss ranged from 0% to 87% of initial litter mass (mean: 23.3%) with an average experimental duration of 124 days. Meanwhile, rates of CO_2_ production from decaying litter ranged from 0.007 to 113.4 mg CO_2_-C g^−1^ (mean: 18.2 mg g^−1^), with an average incubation length of 107 days. The dynamics of litter decay were strongly influenced by microbial community composition, with an average coefficient of variation (*CV*) in decomposition rate of 0.193 ± 0.070 (p = 0.005) across inoculum treatments (Figure 1); this corresponded to cross-treatment variation in litter mass loss of approximately 5% of initial mass (*D*_*ML*_ = 5.40 ± 0.94, p < 0.001), whereas CO_2_ flux varied by about 4 mg CO_2_-C g^−1^ litter across inoculum treatments (*D*_*CO2*_ = 4.00 ± 1.38, p = 0.003). There was no significant difference in *CV* for studies quantifying mass loss (CV = 0.145 ± 0.072) versus CO_2_ flux (CV = 0.328 ± 0.121), and all patterns were similar whether analyses examined *CV, D*_*ML*_, or *D*_*CO2*_ (Figure 1). Microbial inoculum effect sizes were comparable to the variation in decay attributable to litter chemistry; *CV* across litter types was 0.235 ± 0.093 (p = 0.011).

Effect sizes were not significantly different between studies that took place in the field or in the laboratory, between terrestrial and freshwater environments, between studies employing reduced complexity versus whole-community inoculum treatments, or among studies where inocula consisted of bacteria alone, fungi alone, or both together (Figure 1). However, CV of mass loss tended to be lower for field versus laboratory studies (Figure 1). For experiments with reduced complexity microbial communities, *CV* was not correlated with the length of the diversity gradient (i.e., maximum species richness of the inoculum) (β = −0.002, p = 0.620). *CV* was also uncorrelated with the length of decomposition (β = −0.0003, p = 0.126) and the C:N ratio (β = 0.0002, p = 0.393) or lignin content (β = 0.002, p = 0.539) of the litter being decomposed.

**Figure 2:**
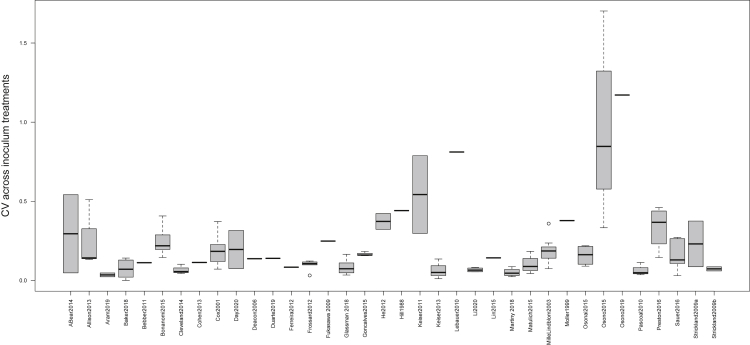
Boxplots indicating mean coefficient of variation (CV) of decomposition across different inoculum treatments in each of the 36 studies included in the meta-analysis Variation within studies reflects the fact that inoculum treatments were compared at different time points and/or across different litter types.

CV showed marked variation among studies (Figure 2). No study-level attribute explained this variation, aside from the number of pairwise comparisons made: there was a weak tendency for studies with a larger number of inoculum treatments to find larger *CV* (β = 0.012, p < 0.001). However, this finding was driven by a single study with 68 inoculum treatments; when this study was removed, the relationship was no longer significant (β = 0.001, p = 0.753). We also explored whether studies that repeatedly measured decomposition had disproportionate influence over observed patterns, as these experiments yielded separate *CV* estimates for each time point. We re-ran all the analyses described earlier, this time calculating a single *CV* value for each litter type treatment within a study (i.e., *CV* estimates integrated across all time points). We saw no significant change in effect sizes calculated in this way, and the overall *CV* was quite similar (0.256 ± 0.152, p < 0.001) (Table S2).

Across the 36 studies analyzed here, six provided data on bacterial community composition and four provided information about fungal communities. There were only two cases in which dissimilarity in bacterial or fungal community composition was correlated with dissimilarity in mass loss rates across inoculum treatments (Table 1).

**Table 1:** Test statistics (*r*) and associated p values (in parentheses, with bold text indicating values < 0.05) for Mantel tests examining correlations between dissimilarity in microbial communities and mass loss patterns within individual studies.

Study	Bacterial communities	Fungal communities
Baker et al. (2018)	−0.516 (0.966)	NA
Cleveland et al. (2014)	0.076 (0.368)	NA
Frossard et al. (2012)	−0.104 (0.654)	**0.517 (0.006)**
Glassman et al. (2018)	**0.757 (0.001)**	0.014 (0.389)
Li et al. (2020)	0.024 (0.297)	−0.079 (0.926)
Preston and Basiliko, (2016)	0.015 (0.377)	0.014 (0.434)

Analyses were conducted separately for bacterial and fungal communities on decomposing litter.

## Discussion

We found strong evidence that differences in the composition of microbial inocula on sterile litter resulted in biologically significant variability in rates of decay. On average, the coefficient of variation due to inoculum type was 20%, translating to an absolute difference of 5% mass loss over an average experimental period of 119 days. Notably, however, the degree of variation explained by inoculum type was uncoupled from any experimental attribute: the diversity and composition of the inoculum, the composition of the litter being decomposed, or the length of observation.

### Hypothesis 1: Diversity-function relationships in litter decay

We did not find evidence that variation in mass loss across different inoculum treatments was contingent upon the taxonomic diversity of those inocula. This result contrasts with evidence showing positive diversity-function relationships in soils (Bell et al., 2005; Wagg et al., 2019). However, it should be noted that the question addressed by our analysis is subtly different from that usually addressed in biodiversity-ecosystem function experiments. We were not investigating whether a process *rate* (in this case, plant litter decay) increased with diversity; rather, we tested whether among-treatment *variation* in decay increased with the length or steepness of the diversity gradient, i.e. number of species in the maximum diversity treatments. We initially hypothesized that such relationships might be easier to detect in experiments with shorter diversity gradients, as diversity-function relationships typically saturate with an increasing number of taxa. Alternatively, however, niche complementarity and/or facilitation among microbial taxa might generate increasingly distinct patterns of litter mineralization in more diverse species mixtures (Qiu and Cardinale, 2020). Yet no significant relationship was found in either direction. Our analysis may not have had sufficient power to fully address the question: only two experiments combined more than 10 species in a given inoculum treatment. Notably, however, effect size metrics did not vary significantly between studies that used “reduced complexity” inocula, consisting of one to 64 species, versus “whole community” inocula, which presumably contained hundreds or thousands of species.

To further explore diversity-function relationships, we also examined evidence for selection effects by regressing variability in decay rates against the number of pairwise treatment comparisons. We found only a weakly positive relationship, which was contingent upon the inclusion of a study with 68 comparisons among individual fungal strains; this is a relatively reassuring finding, suggesting that adding more treatment contrasts to a given experiment did not strongly inflate type I error. Finally, we examined diversity-function relationships at the level of coarse taxonomic groups, finding that microbial community effects were similar when inoculum treatments included fungi, bacteria, or both together. Although prokaryotic communities are more taxonomically diverse than fungal communities (Bahram et al., 2018) and therefore may exhibit a higher level of functional redundancy, we found no evidence that inoculum effects were stronger when manipulating fungal decomposers versus bacteria.

Ultimately, we found that the effect of different microbial inocula on mass loss tended to be equally distinct whether the inocula being compared consisted of single fungal or bacterial strains or whole microbial communities. We speculate that the mechanisms generating structure-function relationships vary depending upon the taxonomic richness of the community under examination. In low-diversity microbial communities, the inclusion of specific taxa with unique functional capabilities may drive variability among different inoculum treatments. In high-diversity scenarios, such as the “whole community” inoculum experiments, the emergence of functionally distinct cross-feeding networks may be more important.

### Hypothesis 2: Effects of experimental duration on microbial community structure-function relationships

The evolution of microbial community structure-function relationships through time is complex because it is underpinned by both community-level processes (immigration, local extinction), individual-level processes (changes in cell physiology and metabolism), and environmental processes (feedbacks between microbial communities and the chemistry of the litter being decomposed). We might expect the composition of decomposer microbial communities to diverge throughout the decay process for several reasons. First, community composition is influenced by stochastic processes that influence dispersal and local extinction, leading to compositional divergence through time (Johansen et al., 2019). In addition, the chemical complexity of litter increases throughout decomposition (Wickings et al., 2012; Lehmann et al., 2020), reflecting the innumerable pathways of microbial anabolism. This chemical diversity might permit greater ecological specialization within microbial communities. As a result of these drivers, we might expect microbial community structure-function effects to strengthen over the course of decay, with higher functional variability among inoculum treatments as time goes on. However, it should be noted that there is evidence for rapid microbial community *convergence* at higher levels of ecological organization (e.g. functional guilds), because a given resource environment shapes cross-feeding networks with predictable metabolic profiles (Estrela et al., 2022); this would tend to reduce functional variation across inoculum treatments through time. However, our data showed no signal of community convergence or divergence: across the studies we examined, we did not find any correlation between the magnitude of microbial inoculum effect sizes and the length of decomposition, which in this dataset ranged up to 540 days.

The absence of any predictable relationship between effect size and time may reflect the relatively small size of our dataset, or it could mean that decay experiments are not conducted over the timescales most relevant to the processes underlying community shifts. We also note that the vast majority (83%) of studies in our dataset restricted immigration of new taxa onto decomposing litter. In the laboratory, inoculum treatments were applied in closed microcosms; in the field, many studies employed a “microbial cage” design where litterbag mesh sizes (0.45–0.2 μm) restrict entry of fungi and most bacteria. Therefore, temporal changes in microbial communities would be restricted to changes in abundance of the taxa present in the original inoculum; this might dampen any shifts in microbial community structure that would occur in an open environment, weakening the correlation between inoculum effect size and time.

### Hypothesis 3: Litter chemistry and its interactions with microbial inoculum effects

Litter chemistry is the dominant control on rates of mass loss in ecosystems worldwide (Zhang et al., 2008), and it also impacts the composition of the microbial communities that colonize plant litter (Bray et al., 2012). Although only a minority of the experiments examined here conducted fully factorial manipulations of both litter type and microbial inoculum type, such experiments are especially valuable because they disentangle the direct and indirect microbially mediated effects of litter chemistry on decomposition. In two of the six such studies examined, the effects of litter type were stronger than those of inoculum type; in the remaining four studies, these effect sizes were not significantly different from one another. This comparison, although admittedly limited in scope, suggests that the importance of the microbial community for predicting rates of litter decay may rival that of the litter chemistry. However, there was little evidence that different chemical attributes of plant litter can predict how strong microbial inoculum effects are likely to be. We found that the influence of inoculum type on decay rate was not linked to the C:N ratio or lignin content of the substrate being decomposed. Although we expected a stronger signature of microbial community structure in lignin-rich and low-nutrient substrates, where decomposition of the dominant litter constituents is taxonomically restricted (De Boer et al., 2005), this was clearly not the case. These indices of litter chemistry may be too simplistic to capture functional specialization within decomposer communities. Alternatively, if microbial communities are specialized to their local litter type (“home field advantage”), then we would not expect a directional relationship between litter chemistry and the influence of inoculum on decomposition.

### Hypothesis 4: Relationship between community dissimilarity and functional dissimilarity

If the composition of microbial communities controls the ecosystem processes they perform, we would expect communities with a larger number of taxa in common to exhibit more similar rates of function. We tested this prediction by examining the correspondence between microbial community dissimilarity and functional dissimilarity within individual studies, finding significant relationships in only two out of ten cases. The scope of our analysis was limited by the availability of community data reported, and our measurement of microbial community dissimilarity (community ordination scores) is imperfect. These scores do not capture all the dimensions of multivariate space across which communities vary, and their numeric values are not comparable across studies. Therefore, we refrain from making broad generalizations based on these data. However, it is clear that within the context of the experiments analyzed here, more taxonomically divergent microbial communities clearly do not necessarily result in more dissimilar rates of function.

### When and where do we need information about microbial communities to predict ecosystem processes?

This meta-analysis of litter inoculation experiments shows conclusively that the composition of microbial communities has substantial impacts on the rate of organic matter decay in the relatively early stages of decomposition. Unfortunately, however, these data do not fully address the question we initially posed: under which circumstances is information about microbial community structure necessary to predict ecosystem process rates? Although there was marked variation in effect sizes across studies, this variability could not be attributed to microbial diversity, litter chemistry, duration of decomposition, or the dissimilarity among decomposer communities. This finding echoes the dramatic idiosyncrasy in microbial community structure-function relationships documented in many previous studies (Mikola et al., 1998; Wardle et al., 1997).

The studies examined here were designed to isolate the effects of different microbial assemblages on litter decay and therefore carefully controlled background variation in climate, litter chemistry, and disturbance; this may explain why we found that effect sizes were larger in laboratory versus field settings. Our analysis also suggests a much more pervasive and substantial influence of microbial communities in comparison to other meta-analyses, which have uncovered relatively weak structure-function relationships (e.g. Rocca et al., 2015; Graham et al., 2016). Those analyses focused largely on correlative studies where variation in microbial communities was documented alongside ecosystem processes and potential confounding factors were not intentionally eliminated. It may be harder to quantify the impacts of microbial communities on decay against the background of real-world environmental variability, but this does not mean that community structure is functionally insignificant.

Our meta-analysis focused on a single ecosystem process, plant litter decomposition, which is carried out by most members of the soil microbial community; structure-function relationships might be even more pronounced for “narrow” biogeochemical processes such as nitrification or methanogenesis. Moreover, a major shortcoming of most litterbag studies is that they cannot provide insight into the fate of organic matter inputs. Mass lost in any given time interval may represent complete mineralization to CO_2_, leaching of dissolved organic material into deeper soil profiles, or incorporation of litter-derived material into various microbial biomolecules. In addition to affecting rates of mass loss, inoculum characteristics may also influence the long-term fate of plant-derived carbon being decomposed. For example, the composition of the microbial community alters its biomass chemistry in ways that influence subsequent mineral stabilization (Neurath et al., 2021; Whitman et al., 2018). Therefore, the strong influence of microbial inocula on litter decay rate, which could be observed even at the earliest stages of decomposition, may be further amplified by variation in the longer-term fate of the microbial residues.

### Limitations of the study

Our findings leave us in a quandary whereby we must acknowledge that microbial community composition can strongly shape soil biogeochemistry, but we cannot predict which features of the soil microbiome are the dominant controls on process rates. Moreover, the methods used to identify microbial taxa from sequence data yield context-dependent definitions of individual “species,” often precluding direct comparisons across studies (Callahan et al., 2017). In response to these dilemmas, Morris et al. (2020) have advocated a “taxonomically agnostic” approach, searching for structure-function correlations that may occur across multiple levels of biological organization (functional genes, species, or groups of interacting taxa). The discovery that soil bacterial and fungal communities follow predictable biogeographic patterns, are dominated by a subset of globally distributed taxa (Bahram et al., 2018; Delgado-Baquerizo et al., 2018; Tedersoo et al., 2014), and self-assemble into stable cross-feeding networks (Pascual-García et al., 2020) can also help focus future research efforts on particular species or functional guilds. Future litter inoculation experiments should provide information about the taxonomic and functional makeup of microbial communities to permit more rigorous identification of these potential keystone taxa or functional groups. Ultimately, the strong microbial community structure-function relationships demonstrated here suggest that we cannot safely ignore microbial ecological interactions when attempting to predict or manage soil biogeochemical cycles.
