# Supplementary material for: A quantitative analysis of microbial community structure-function relationships in plant litter decay
Source: iScience. 2022 Jun 3;25(7):104523. doi: 10.1016/j.isci.2022.104523 (PMC9218368; doi:10.1016/j.isci.2022.104523)
Supplement: Document S1. Tables S1 and S2 [file mmc1.pdf]

iScience, Volume *25*

Supplemental information

A quantitative analysis of microbial community structure-function relationships in plant litter decay

Bonnie Waring, Anna Gee, Guopeng Liang, and Savannah Adkins

Table S1. Bibliographic information for publications included in the meta-analysis (related to Figure 1 and Figure 2)

Authors	Source Title	Publication Year	DOI
A'Bear, AD; Boddy, L; Kandeler, E; Ruess, L; Jones, TH	SOIL BIOLOGY & BIOCHEMISTRY	2014	10.1016/j.soilbio.2014.05.031
Allison, SD; Lu, Y; Weihe, C; Goulden, ML; Martiny, AC; Treseder, KK; Martiny, JBH	ECOLOGY	2013	10.1890/12-1243.1
Aram, K; Rizzo, DM	FORESTS	2019	10.3390/f10050434
Baker, NR; Khalili, B; Martiny, JBH; Allison, SD	ECOLOGY	2018	10.1002/ecy.2345
Bebber, DP; Watkinson, SC; Boddy, L; Darrah, PR	OECOLOGIA	2011	10.1007/s00442-011-2057-2
Bonanomi, G; Capodilupo, M; Incerti, G; Mazzoleni, S; Scala, F	COMMUNITY ECOLOGY	2015	10.1556/168.2015.16.2.4
Cleveland, CC; Reed, SC; Keller, AB; Nemergut, DR; O'Neill, SP; Ostertag, R; Vitousek, PM	OECOLOGIA	2014	10.1007/s00442-013-2758-9
Cohen, JS; Blossey, B	AQUATIC ECOLOGY	2013	10.1007/s10452-013-9455-y
Cox, P; Wilkinson, SP; Anderson, JM	BIOLOGY AND FERTILITY OF SOILS	2001	10.1007/s003740000315
Day, NJ; Cumming, SG; Dunfield, KE; Johnstone, JF; Mack, MC; Reid, KA; Turetsky, MR; Walker, XJ; Baltzer, JL	FRONTIERS IN FORESTS AND GLOBAL CHANGE	2020	10.3389/ffgc.2020.00068
Deacon, LJ; Pryce-Miller, EJ; Frankland, JC; Bainbridge, BW; Moore, PD; Robinson, CH	SOIL BIOLOGY & BIOCHEMISTRY	2006	10.1016/j.soilbio.2005.04.013
Duarte, S; Antunes, B; Trabulo, J; Seena, S; Cassio, F; Pascoal, C	FUNGAL ECOLOGY	2019	10.1016/j.funeco.2019.06.001
Ferreira, V; Chauvet, E	AQUATIC MICROBIAL ECOLOGY	2012	10.3354/ame01556
Frossard, A; Gerull, L; Mutz, M; Gessner, MO	ENVIRONMENTAL MICROBIOLOGY	2012	10.1111/j.1462-2920.2012.02865.x

Fukasawa, Y; Osono, T; Takeda, H	ECOLOGICAL RESEARCH	2009	10.1007/s11284-009-0582-9
Glassman, SI; Weihe, C; Li, JH; Albright, MBN; Looby, CI; Martiny, AC; Treseder, KK; Allison, SD; Martiny, JBH	PROCEEDINGS OF THE NATIONAL ACADEMY OF SCIENCES OF THE UNITED STATES OF AMERICA	2018	10.1073/pnas.1811269115
Goncalves, AL; Graca, MAS; Canhoto, C	FUNGAL ECOLOGY	2015	10.1016/j.funeco.2015.05.013
He, XB; Han, GM; Lin, YH; Tian, XJ; Xiang, CG; Tian, QJ; Wang, FY; He, ZH	ECOLOGICAL RESEARCH	2012	10.1007/s11284-011-0898-0
HILL, NM; PATRIQUIN, DG	SOIL BIOLOGY & BIOCHEMISTRY	1988	10.1016/0038-0717(88)90143-5
Keiser, AD; Knoepp, JD; Bradford, MA	PLANT AND SOIL	2013	10.1007/s11104-013-1730-0
Keiser, AD; Strickland, MS; Fierer, N; Bradford, MA	BIOGEOSCIENCES	2011	10.5194/bg-8-1477-2011
LeBauer, DS	CANADIAN JOURNAL OF FOREST RESEARCH	2010	10.1139/X10-054
Li, YB; Veen, GF; Hol, WHG; Vandenbrande, S; Hannula, SE; ten Hooven, FC; Li, Q; Liang, WJ; Bezemer, TM	SOIL BIOLOGY & BIOCHEMISTRY	2020	10.1016/j.soilbio.2020.107783
Lin, YH; He, XB; Ma, TW; Han, GM; Xiang, CG	PEDOBIOLOGIA	2015	10.1016/j.pedobi.2015.09.001
Martiny, JBH; Martiny, AC; Weihe, C; Lu, Y; Berlemont, R; Brodie, EL; Goulden, ML; Treseder, KK; Allison, SD	ISME JOURNAL	2017	10.1038/ismej.2016.122
Matulich, KL; Martiny, JBH	ECOLOGY	2015	10.1890/14-0357.1
Mille-Lindblom, C; Tranvik, LJ	MICROBIAL ECOLOGY	2003	10.1007/s00248-002-2030-z
Moller, J; Miller, M; Kjoller, A	SOIL BIOLOGY & BIOCHEMISTRY	1999	10.1016/S0038-0717(98)00138-2
Osanai, Y; Janes, JK; Newton, PCD; Hovenden, MJ	SOIL BIOLOGY & BIOCHEMISTRY	2015	10.1016/j.soilbio.2015.02.032
Osono, T	JOURNAL OF FOREST RESEARCH	2015	10.1007/s10310-014-0462-1
Osono, T	ECOLOGICAL RESEARCH	2020	10.1111/1440-1703.12063
Pascoal, C; Cassio, F; Nikolcheva, L; Barlocher, F	MICROBIAL ECOLOGY	2010	10.1007/s00248-009-9567-z

Preston, MD; Basiliko, N	GEOMICROBIOLOGY JOURNAL	2016	10.1080/01490451.2014.999293
Sauer, FG; Bundschuh, M; Zubrod, JP; Schafer, RB; Thompson, K; Kefford, BJ	AQUATIC TOXICOLOGY	2016	10.1016/j.aquatox.2016.06.014
Strickland, MS; Lauber, C; Fierer, N; Bradford, MA	ECOLOGY	2009	10.1890/08-0296.1
Strickland, MS; Osburn, E; Lauber, C; Fierer, N; Bradford, MA	FUNCTIONAL ECOLOGY	2009	10.1111/j.1365-2435.2008.01515.x

Table S2. Results of sub-group analysis (effect sizes and 95% confidence intervals) when mass loss values within an inoculum treatment were averaged across time points in each individual study (compare to results in Figure 1).

Grouping factor	Level	Mean effect size (CV)	95% CI
Domain	Aquatic	0.035	-0.009, 0.078
	Terrestrial	0.351	0.309, 0.392
Experimental context	Field	0.241	-0.891, 1.374
	Laboratory	0.383	-0.388, 1.54
Inoculum type	Reduced complexity	0.214	0.078, 0.421
	Whole community	0.300	0.089, 0.510
Inoculum Taxonomic composition	Bacteria-only	0.310	-1.612, 2.236
	Fungi-only	0.215	0.006, 0.424
	Mixed inoculum	0.297	0.087, 0.509
